# Particulate matter on foliage of *Betula pendula*, *Quercus robur*, and *Tilia cordata*: deposition and ecophysiology

**DOI:** 10.1007/s11356-020-07672-0

**Published:** 2020-01-13

**Authors:** Adrian Łukowski, Robert Popek, Piotr Karolewski

**Affiliations:** 1 Faculty of Forestry, Poznań University of Life Sciences, Wojska Polskiego 71c, 60-625 Poznań, Poland; 2 Institute of Dendrology Polish Academy of Sciences, Parkowa 5, 62-035 Kórnik, Poland; 3 Section of Basic Research in Horticulture, Department of Plant Protection, Institute of Horticultural Sciences, Warsaw University of Life Sciences - SGGW, Nowoursynowska 159, 02-776 Warsaw, Poland

**Keywords:** Birch, Chlorophyll *a* fluorescence, Dust, Epicuticular waxes, Gas exchange, Lime, Oak

## Abstract

Trees in urban and industrial areas significantly help to limit the amount of particulate matter (PM) suspended in the air, but PM has a negative impact on their life. The amount of PM gathered on leaves depends on quantity, size, and morphology of leaves and can also be increased by the presence of epicuticular waxes, in which PM can become stuck or immersed. In this study, we determined the ability of PM to accumulate on leaves in relation to the species of tree and PM source. We tested saplings of three common European tree species (*Betula pendula*, *Quercus robur*, and *Tilia cordata*) by experimentally polluting them with PM from different sources (cement, construction, and roadside PM), and then assessing the effects of PM on plant growth and ecophysiology. In all studied species, we have found two types of PM accumulation: a layer on the leaf surface and an in-wax layer. Results showed that the studied species accumulate PM on their leaf blade, reducing the efficiency of its photosynthetic apparatus, which in a broader sense can be considered a reduction in the plants’ normal functioning. Saplings of *Q. robur* suffered the least, whereas *B. pendula* (especially photosynthetic rate and conductivity) and *T. cordata* (especially increase in leader shoot length) exhibited greater negative effects. The foliage of *B. pendula* collected the most PM, followed by *Q. robur*, and then *T. cordata*, regardless of the dust’s source. All tested species showed a tendency for higher wax production when growing under PM pollution stress. We believe that, potentially, *B. pendula* best enhances the quality of the PM-contaminated environment; however, faster leaf fall, reduced productivity, and worse quality of wood should be considered in urban forest management.

## Introduction

Consumerism and industrialisation have given birth to air pollution, an important global problem with a crucial impact on the health and lives of people (Rai 2016; Khaniabadi et al. 2017). Studies have emphasised that of the many types of pollutants, one of the most dangerous components of air pollution to human health is particulate matter (PM), a mixture of liquid and solid particles that are suspended in the air (Bell et al. 2011; Weuve 2012). In atmospheric pollution research, these pollutants are divided according to their aerodynamic diameter, for instance, PM_10_ (particles equal or less than 10 μm) and PM_2.5_ (equal or less than 2.5 μm). However, in biological research, particles are usually divided into four categories based on size ranges: large PM (10–100 μm), coarse PM (2.5–10 μm), fine PM (0.1–2.5 μm), and ultra-fine PM (< 0.1 μm; Beckett et al. 1998; Dzierżanowski et al. 2011; Power et al. 2009). PM is formed by both natural forces (volcanic activity, forest fires, geochemical processes, etc.) and human activity (Ferreira-Baptista and De Miguel 2005). PM of human origin, however, is frequently enriched by polycyclic aromatic hydrocarbons, heavy metals, and other toxic substances (Jouraeva et al. 2002; Alghamdi 2016; Popek et al. 2017a). Industrial and urban areas, as well as areas with high traffic, are characterised as having the highest levels of PM contamination (Janssen et al. 1997; Popek et al. 2015). It is estimated that the current level of anthropogenic PM contamination causes approximately two million premature deaths annually worldwide (Silva et al. 2013). This is why it is so important to study and use all safe, available, affordable methods to prevent and effectively eliminate the source of this complex problem.

If pollutants have been released into the atmosphere, in addition to technical equipment such as chimney and car particle filters, plants, especially deciduous trees and shrubs, have an important role in reducing the amount of PM in the atmosphere by accumulating pollutants on the surfaces of their leaves (Popek et al. 2017a). Plants growing in urban environments improve air quality and serve as natural filters for PM (Popek et al. 2015; Przybysz et al. 2014; Qiu et al. 2009; Sæbø et al. 2012), but species differ in their ability to accumulate PM (Dzierżanowski et al. 2011; Popek et al. 2017b; Song et al. 2015). Deciduous trees, due to their large leaf surface area and wide distribution, possess the greatest ability to accumulate PM, although only in the growing season (McDonald et al. 2007). The use of trees as air bioindicators is becoming more and more widespread, and there is even a belief that trees will displace lichen in this role, because of the greater availability of biological material; simplicity of species identification, sampling, and treatment; and ubiquity of some tree taxa (Rai 2016). In contrast, technical equipment, such as electrostatic precipitators and filters, more efficiently reduces dust emissions, but only for certain point sources. It can be expected that the leaves of trees can accumulate a significant amount of PM and accompanying toxins, which on the scale of a whole city can be counted in tonnes (Nowak et al. 2006). The amount of PM gathered on leaves depends on quantity, size, and morphology of leaves (Prusty et al. 2005) and can also be increased by the presence of epicuticular waxes, in which PM can become stuck or immersed (Dzierżanowski et al. 2011; Leonard et al. 2016).

Trees in urban and industrial areas significantly help to limit the amount of PM suspended in the air (McDonald et al. 2007). In Beijing (China), only in 1 year, trees in the city centre can remove 772 t of PM from the air (Yang et al. 2005). Moreover, as shown in the last estimations of Kroeger et al. (2018), reforestation of 27 US cities to 20% of their land area can lower average annual PM_10_ concentration by > 2 μg/m^3^ and average daily summer temperatures by > 2 °C, which can result in an estimated savings of about $102 million. However, the research of Pugh et al. (2012) that shows that vegetation plays the most important role at short distances from the source of PM emission; planting trees and shrubs along the roads can reduce concentrations of PM even by 60%. Nevertheless, PM has a negative impact on their life (Farmer 1993). All types of air pollutants, especially PM, have potentially harmful impacts on biochemical parameters, which can further lead to a reduction in the overall growth and development of plants (Naidoo and Chirkoot 2004; Nanos and Ilias 2007; Rai 2016). For example, PM from cement kilns decreases height, biomass, net productivity, and chlorophyll content in plants (Prasad and Inamdar 1990). Plants that are constantly exposed to PM successively accumulate and integrate these pollutants into their leaves, which can cause direct chronic injury (e.g. chlorosis) and loss of productivity (e.g. premature dying of leaves, reduction in height increase). Conversely, many plants do not show visible changes, because much more often, changes in the plant occur at the anatomical and biochemical level (Rai 2016). Indeed, it appears that PM mainly decreases plant vigour via a shading effect, whereby accumulated PM absorbs and scatters light rays, preventing them from freely accessing chloroplasts, which is reflected in a decline in the efficiency of photosynthesis (Przybysz et al. 2014; Saadullah et al. 2014; Popek et al. 2017b) and an additional increase in leaf temperature (Eller 1977; Hirano et al. 1995; Naidoo and Chirkoot 2004), or decrease, due to effects on albedo (Glenn et al. 2001; Sharma et al. 2015). Many papers have reported the impacts of anthropogenic stress factors on the photosynthetic apparatus, but only some of them have considered PM as an important variable in reducing the efficiency of this process (Hirano et al. 1995; Naidoo and Chirkoot 2004; Nanos and Ilias 2007; Przybysz et al. 2014; Popek et al. 2017b). Moreover, PM can be deposited into the openings of the stomata and disturb the intensity of transpiration, or even penetrate into the stomatal pores and disturb mesophyll function (Burkhardt and Grantz 2016; Grantz et al. 2018). Furthermore, high levels of PM pollution may also reduce the amount of chlorophyll in plant cells (Prusty et al. 2005), and the fluorescence of chlorophyll *a* may be altered (Naidoo and Chirkoot 2004; Popek et al. 2017b). The impacts of PM can be far-reaching because their accumulation has significant effects not only on the physiological processes of the entire plant but also on organisms in upper trophic levels, such as folivorous insects (Khan et al. 2013; Łukowski et al. 2018).

There are two opposite views regarding the impact of PM on urban forests. The one view holds that PM may harmfully affect the health and biodiversity of urban forests (Rai 2016), whereas the other posits that deposition of PM could serve as a source of nutrients (Cape 2008). Thus, not enough is currently known regarding the impact of PM on deposition patterns, plant growth and development, and ecophysiology, nor concerning the importance of biomonitoring many important and widespread forest tree species. It is undeniable that the adverse or beneficial effects of dust depend on the amount and origin (source) of the dust, and both of these factors determine its potential toxicity.

The aim of the study was to determine the influence of PM accumulation on the photosynthetic processes and wax layer and how this varied according to tree species and the source of PM, under controlled conditions. We tested three tree species (*Betula pendula* Roth., *Quercus robur* L., and *Tilia cordata* Mill.) by experimentally polluting them with PM from different sources (cement, construction, and roadside PM), and then, we assessed the effects of PM on plant growth and ecophysiology. The species we choose are characterized by widespread occurrence in European managed and urban forests. The specific objectives of the research were to (a) check the accumulation of two types, _S_PM (surface PM) and _W_PM (in-wax PM), and three size fractions (10–100, 2.5–10, and 0.2–2.5 μm) of PM on foliage; (b) investigate the amount of epicuticular waxes on the surfaces of leaves; (c) assess the efficiency of the photosynthetic process; (d) study the effect of dust on annual increases in leader shoot length; and (e) correlate the efficiency of the photosynthetic apparatus and plant growth with the amount of PM pollution. Many previous studies on the ecophysiology and selection of trees and shrubs for contaminated areas have been carried out under field conditions with an unspecified and often variable type of dust, or a specific mixture characteristic only to the study location (Jouraeva et al. 2002; Song et al. 2015; Nguyen et al. 2015). We proposed a different approach; namely, in controlled conditions, we used dust (with specific origin and fractional composition) to investigate how various PM sources affect the ecophysiology and growth of trees. We hypothesise that (1) differences in PM accumulation capacity between the tested species are significant, (2) the wax layer on the leaf blades of the studied species plays an important role in the absorption of PM, (3) PM has a negative impact on the photosynthetic apparatus, and (4) PM also has a negative impact on annual increases in leader shoot length.

## Materials and methods

### Plant material and study area

Studies were conducted in the vegetation seasons of 2015 and 2016, using *Betula pendula* Roth., *Quercus robur* L., and *Tilia cordata* Mill. growing in pots filled with the same substrate (forest soil from mature oak/pine forest mixed with neutralised peat in a 1:1 ratio). All data from both seasons were taken into consideration; therefore, we considered ‘year’ to be a random factor in our statistical model. All trees chosen for the experiment were the same age (5 years old), were ca. 100–120 cm in height, and were in good condition (healthy and free from pests). To protect plants from outside stress factors, especially the accumulation of PM from the atmosphere and the possibility of rinsing away dust used in the experiment via rain, trees were placed in greenhouses under appropriate growing conditions. Plants were divided into four groups of four plants of each species and placed in separate greenhouses (*n* = 4 replications for one tree species and one PM source). Each year, the trees in the first three groups were dusted with equal volumes (5 cm^3^) of either (i) construction PM, (ii) cement PM, or (iii) roadside PM, consisting of particles less than 100 μm in diameter. For this purpose using a sieve (< 100 μm), we evenly spread approx. 1.25 cm^3^ of dust directly over the top of the individual plant, once at the earliest possible date for mature leaves (mid-May). Precise delivery of dust on the leaves is difficult and as we were aware of the possibility of unequal delivery of dust in our study, we carried out the study in two growing seasons. In addition, this procedure was always performed by the same person. Cement PM consisted of Portland cement particles (Cemmas A.S., Poland), roadside PM was collected from the edge of a busy road (Matyi and Bolesława Krzywoustego streets) in Poznań, Poland (52° 24′ N, 16° 56′ E), and construction PM was collected from the debris of various demolished buildings in the city. Cement was purchased but roadside PM and construction PM were collected from the surface of their excessive accumulation in the city. All sources were sieved prior to the experiment (we used to isolate particles less than 100 μm in diameter). Mass-based ratios of particle size fractions in the applied dust were 90:7:3 for construction PM, 93:5:2 for cement PM, and 86:12:2 for roadside PM, using large (10–100 μm), coarse (2.5–10 μm), and fine (0.2–2.5 μm) fractions, respectively. The fourth group, consisting of control trees, was not subjected to any stress conditions. In greenhouses, the average daily temperature during the growing season (mid-May–mid-October) was 18.1 °C (± 0.6). All plants were watered as necessary.

### Analysis of accumulation of PM and epicuticular waxes on leaves

#### Sample collection

Leaves were harvested from trees (*n* = 96 samples) at the end of each growing season (*n* = 4 PM sources × 2 years × 3 species × 4 trees). Samples consisted of leaves gathered from different branches of crowns, making them representative of each tree. Leaves (only leaf blades without petioles) were placed in paper bags, labelled, and kept at ambient temperature until analysis. To obtain sufficient material to determine the fine fraction of PM and still avoid filter blockage by particles during filtration, the leaf area per sample ranged from 300 to 400 cm^2^.

#### Quantitative analysis of PM and epicuticular waxes

The amount of PM and epicuticular waxes on leaves were determined gravimetrically as described in detail by Dzierżanowski et al. (2011), in two categories: surface PM (_S_PM), which was washed off with water, and in-wax PM (_W_PM), which was washed off with chloroform. Leaf samples were hand washed, firstly with 250 ml of distilled water (conductivity level ~ 10 μS/cm) and then with 150 ml of chloroform each for 1 min. The method of hand washing of leaves used has one small disadvantage: there is no certainty that the intensity of mixing is always the same. In order to minimize this limit, this type of work was always done by the same person. On the next step, liquids were filtered using a metal sieve (Haver and Boecker, Germany) with a mesh diameter of 100 μm in order to eliminate particles larger than 100 μm. Then, liquids were filtered using pre-weighed type 91 and type 42 paper filters and PTFE membrane filters (Whatman, UK), with pore sizes of 10 μm, 2.5 μm, and 0.2 μm, respectively, what at the end gave three size fractions (10–100, 2.5–10, and 0.2–2.5 μm). To remove the electrostatic charge from the membranes, every time before weighting, all filter were passed through a deioniser gate (HAUG, Switzerland). Moreover, filters before and after filtration were dried in an oven at 60 °C for 30 min in a KCW-100 drying chamber (PREMED, Poland) and stabilised in a closed room, with controlled humidity and temperature for another 30 min. After filtration, all filters were weighed again using the analytical balance Sartorius CP225D (Göttingen, Germany). The quantity of epicuticular waxes was weighed after evaporation of the chloroform collected in pre-weighed beakers. Afterwards, the filtration area of leaves from each sample was measured using a scanner and WinFOLIA 2013 measuring software (Regent Instruments Inc., Canada). The amounts of PM from filters and waxes were then recalculated to μg cm^−2^ of leaves, in order to easily compare different PM sources and species. The insoluble particles are taken into account in these studies.

### Evaluation of efficiency of photosynthetic apparatus

Measurements were carried out four times in each of the 2 years of the study after dust application, and at monthly intervals throughout the vegetation season (in mid-June, mid-July, mid-August, and mid-September). The following parameters of efficiency of the photosynthetic apparatus were measured in vivo.

#### Plant gas exchange

As part of plant gas exchange, measurements of photosynthetic rate and stomatal conductance (*n* = 1920 for each) were evaluated via the LI-6400 Photosynthesis System equipped with the 6-cm^2^ leaf chamber, using an infrared gas analyser method (LI-COR Inc., Lincoln, Nebraska, USA). The light intensity for all measurements was 1500 mmol m^−2^ s^−1^, CO_2_ concentration in the chambers was adjusted to a constant 400 μmol mol^−1^, while relative humidity was approximately 30–35%, and temperature in the leaf chamber varied between 24 and 26 °C. Measurements were conducted during cloudless weather, between 9 a.m. and 1 p.m. For each tree, measurements were conducted on each of five random leaves selected from the middle parts of annual twigs. Each leaf was placed separately in the leaf chamber and maintained until the photosynthesis rate stabilised, when a measurement was recorded. In every month of the study period, leaves were selected for measurement from the same part of the plant (*n* = 4 PM sources × 3 species × 2 years × 4 months × 4 trees × 5 leaves).

#### Determination of chlorophyll *a* fluorescence

On the same leaf samples used for the gas exchange study (*n* = 4 PM sources × 3 species × 2 years × 4 months × 4 trees × 3 leaves), measurements of chlorophyll *a* fluorescence (*n* = 1152) were performed using the portable chlorophyll fluorometer OS1p (Opti-Sciences, Inc., Hudson, New Hampshire, USA). Thirty minutes prior to the measurement, dark adaptation clips were placed on the leaves and the shutter was closed. Measurements of minimal (F_o_) and maximal (F_m_) fluorescence of chlorophyll *a* were obtained using a pulse of high-intensity (3000 μmol m^−2^ s^−1^) light. Maximum quantum efficiency of photosystem II (F_v_/F_m_, where F_v_ is the difference between F_m_ and F_o_) was calculated by the instrument’s software.

### Growth

Measurements of annual increase in leader shoot length (*n* = 96) were performed using a tape measure for all trees used in this study (*n* = 4 PM sources × 3 species × 2 years × 4 trees). In September of each study year, when seasonal growth of tree shoots had been completed, measurements were taken of the increase in the length of the leader shoot of each sapling (formed in the current season), and the average was calculated.

### Statistical analysis

Two-way ANOVA with random effects was conducted to compare values of measured amounts of particulate matter (total, _S_PM, _W_PM, and in three size fractions), rates of photosynthesis, stomatal conductance, maximum quantum efficiency of photosystem II, and annual increase in leader shoot length among tree species and PM sources. ‘Sapling’, nested within ‘plant species’ and ‘PM source’, and ‘year’ (growing season) were included in the model as a random effect. For analysis of plant gas exchange and chlorophyll *a* fluorescence, an additional random effect was added to the model (i.e. ‘leaf’ nested within ‘sapling’ and ‘month’). The expected mean square for a model effect (EMS) rather than restricted maximum likelihood (REML) method was applied to provide detailed information about random effects in Table 1. We used *t* tests to compare _S_PM and _W_PM. Non-parametric Spearman’s correlation coefficients were calculated between different measured variables. Tukey’s HSD test (*P* = 0.05) was employed to assess the significance of differences among variants. Before all analyses, normal distribution was verified with the Shapiro-Wilk test. The data are given as means with standard errors of the mean (± SE). Bold values indicate statistical significance (*P* < 0.05). Statistical analyses were conducted in JMP 13.0 Pro (SAS Institute Inc., Cary, NC, USA).

**Table 1:** Amounts of particulate matter (PM) from different sources—divided into categories of surface PM (_S_PM), in-wax PM (_W_PM), and three size fractions—and amounts of epicuticular waxes on leaves of *Betula pendula*, *Quercus robur*, and *Tilia cordata*. Analysis of variance (ANOVA) was used to assess the statistical significance of differences between PM sources. ‘Sapling’, nested in ‘plant species’ and ‘PM source’, and ‘year’ (growing season) were included in the model as random effects. Bold values indicate *P* < 0.05.

Species	PM source		PM size fraction (μg cm^−2^) (mean ± SE)						Total _S_PM (μg cm^−2^) (mean ± SE)		Total _W_PM (μg cm^−2^) (mean ± SE)		Epicuticular waxes (μg cm^−2^) (mean ± SE)	
			10–100 (μm)		2.5–10 (μm)		0.2–2.5 (μm)							
*Betula pendula*	Control		24.69 ± 1.57		12.00 ± 0.67		4.68 ± 0.32		19.72 ± 0.79		21.64 ± 1.78		588.76 ± 18.68	
	Cement		104.08 ± 2.36		24.83 ± 1.91		10.34 ± 0.71		74.32 ± 2.86		64.93 ± 2.54		784.95 ± 31.27	
	Construction		74.61 ± 1.28		27.53 ± 1.38		9.02 ± 0.24		56.17 ± 1.02		54.99 ± 1.97		777.42 ± 15.58	
	Roadside		84.36 ± 2.56		21.66 ± 1.09		9.56 ± 0.46		64.41 ± 2.33		51.17 ± 1.74		795.65 ± 29.49	
*Quercus robur*	Control		15.79 ± 0.40		6.88 ± 0.25		2.66 ± 0.23		14.83 ± 0.31		10.51 ± 0.49		66.87 ± 2.89	
	Cement		78.02 ± 3.00		13.64 ± 0.59		6.08 ± 0.21		64.75 ± 2.05		32.98 ± 1.44		73.19 ± 3.49	
	Construction		74.66 ± 2.02		19.96 ± 0.96		6.76 ± 0.22		69.50 ± 2.29		31.87 ± 1.28		77.57 ± 3.40	
	Roadside		73.35 ± 1.72		17.18 ± 1.11		6.05 ± 0.26		65.83 ± 2.54		30.75 ± 0.85		76.81 ± 4.65	
*Tilia cordata*	Control		15.48 ± 0.69		5.21 ± 0.40		2.34 ± 0.41		14.99 ± 0.60		8.04 ± 0.41		33.36 ± 2.07	
	Cement		52.54 ± 2.16		19.44 ± 0.75		8.82 ± 0.40		51.25 ± 1.52		29.55 ± 1.21		45.88 ± 1.23	
	Construction		45.25 ± 1.45		11.50 ± 0.37		5.18 ± 0.22		35.39 ± 1.34		26.54 ± 0.58		40.47 ± 1.88	
	Roadside		63.10 ± 2.10		16.81 ± 0.49		7.11 ± 0.29		59.55 ± 2.38		27.47 ± 0.70		45.25 ± 1.05	
ANOVA	d.f.	error	F	*P*	F	*P*	F	*P*	F	*P*	F	*P*	F	*P*
Species	2	47	417.05	**< 0.0001**	193.19	**< 0.0001**	142.13	**< 0.0001**	155.81	**< 0.0001**	777.81	**< 0.0001**	10,430	**< 0.0001**
PM source	3	47	1195.19	**< 0.0001**	226.45	**< 0.0001**	213.75	**< 0.0001**	969.92	**< 0.0001**	529.14	**< 0.0001**	66.73	**< 0.0001**
Species × PM source	6	47	54.89	**< 0.0001**	28.55	**< 0.0001**	12.41	**< 0.0001**	45.40	**< 0.0001**	27.51	**< 0.0001**	49.91	**< 0.0001**
Sapling (species; PM source) and random	36	47	0.67	0.8899	0.55	0.9690	0.44	0.9944	0.44	0.9938	0.62	0.9311	0.21	0.9999
Year and random	1	47	39.56	**< 0.0001**	33.59	**< 0.0001**	1.59	0.2133	20.43	**< 0.0001**	37.61	**< 0.0001**	6.79	**0.0122**

## Results

### Accumulation of PM and epicuticular waxes

**Fig. 1:**
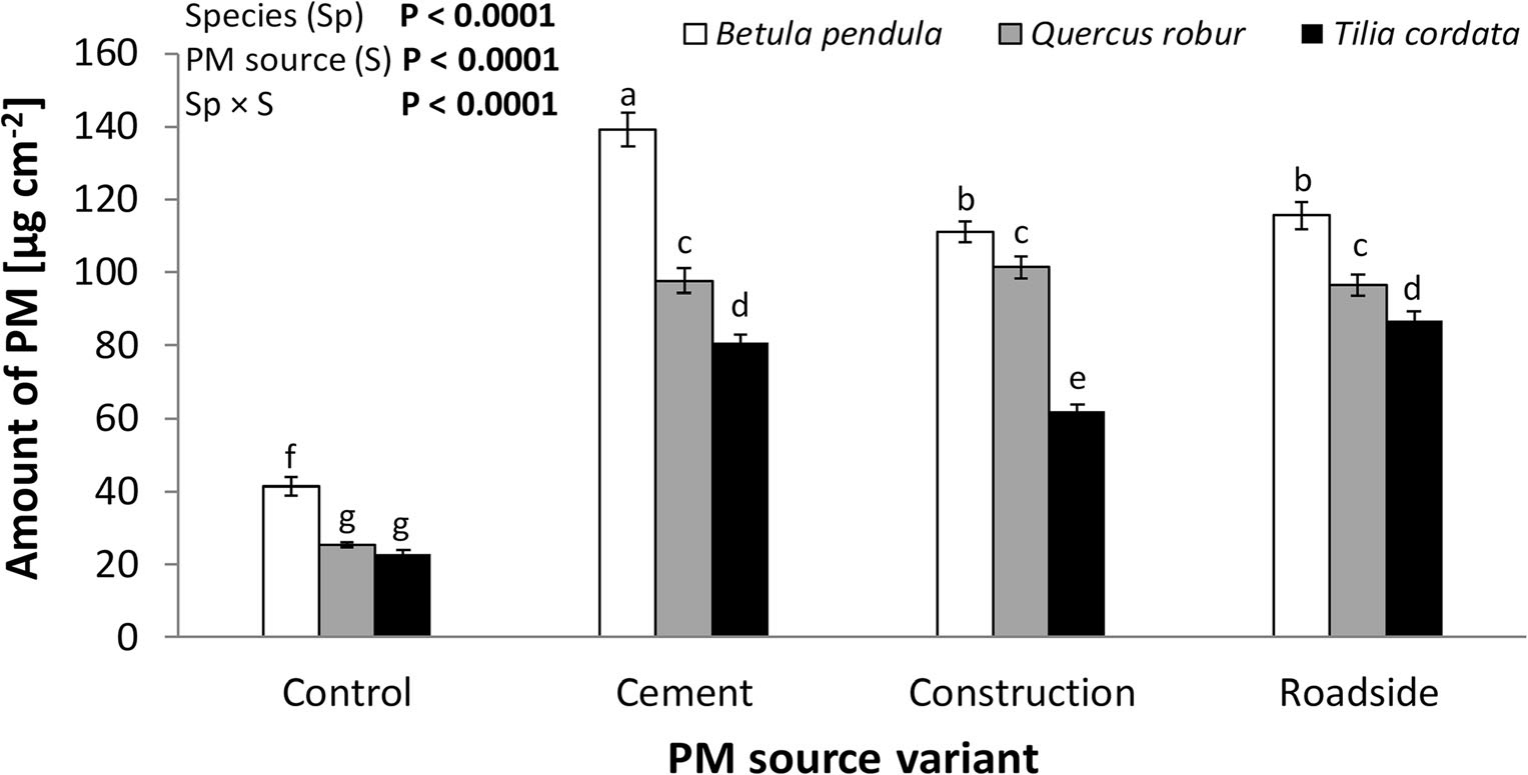
Mean total amount of particulate matter (PM) from different sources deposited on leaves of *Betula pendula*, *Quercus robur*, and *Tilia cordata*. Analysis of variance (ANOVA) was used to assess the statistical significance of PM source, and Tukey’s HSD test (*P* = 0.05) was employed to assess the significance of differences among PM sources. Levels not connected by the same letter are significantly different. ‘Sapling’, nested in ‘plant species’ and ‘PM source’, and ‘year’ (growing season) were included in the model as random effects. Bold values indicate *P* < 0.05. The data are given as means with standard errors of the mean (± SE). Sample size: *n* = 96 samples (see the ‘Materials and methods’ section)

On all saplings tested, PM of both types (_S_PM and _W_PM) and all three size fractions was found. Species and PM source had a significant influence on total PM accumulation. *B. pendula* accumulated approximately 61% (statistical class a in the Tukey’s HSD test; Fig. 1), and *Q. robur* 27% (b) more PM than did *T. cordata* (c). In comparison with control plants (d; Tukey HSD test), leaves of saplings dusted with cement PM accumulated approximately 2.5-fold more PM (a), saplings dusted with roadside PM accumulated 2.3-fold more PM (b), and saplings dusted with construction PM accumulated 2.1-fold more PM (c). Statistical analysis showed interactions among several of the abovementioned effects (Fig. 1). Additionally, for all plant species and PM sources (except *B. pendula* in control and construction; *t* test), more PM was deposited on the leaf surface than was immobilised in waxes (Table 1). Species and PM source had significant effects on the amount of _S_PM and _W_PM on leaves. Regardless of the source of the PM, approximately 53% of total PM was _S_PM and 47% was _W_PM in *B. pendula*, with 67% and 33%, respectively, in *Q. robur*, and 64% and 36%, respectively, in *T. cordata*. Irrespective of plant species, approximately 55% of total PM was _S_PM and 45% was _W_PM on control plant leaves, with 60% and 40%, respectively, in the cement PM group, 59% and 41%, respectively, in the construction PM group, and 63% and 37%, respectively, in the roadside PM group. The amounts of waxes on leaves differed significantly with respect to species and PM source (Table 1). *Betula pendula* had 16.9-fold more (a; Tukey HSD test) and *Q. robur* had 78% more (b) waxes than did *T. cordata* (c). In comparison with control plants (d; Tukey HSD test), leaves of saplings dusted with roadside PM produced approximately 33% more waxes (a), saplings dusted with cement PM produced 31% more waxes (b), and saplings dusted with construction PM produced 30% more waxes (c).

Of all the size fractions measured, regardless of species and dust source, the majority (72%) were large PM (10–100 μm), followed by 20% coarse (2.5–10 μm) and 8% fine PM (0.2–2.5 μm; Table 1). These values differ from the initial values of the dust used in the experiment, as the proportion of large PM decreased in favour of smaller fractions (see the ‘Plant material and study area’ section). Plant species and PM source had significant effects on PM accumulation on leaves in all size fractions. *Betula pendula* accumulated approximately 63% more (a; Tukey HSD test) and *Q. robur* 37% more (b) large PM than did *T. cordata* (c). In all three species, when compared with control plants (d; Tukey HSD test), the leaves of saplings dusted with cement PM accumulated approximately 4.2-fold more PM (a), saplings dusted with roadside PM accumulated 4-fold more PM (b), and saplings dusted with construction PM accumulated 3.5-fold more PM (c). In the case of coarse PM, *B. pendula* accumulated approximately 62% more (a; Tukey HSD test) than did either *T. cordata* (b) or *Q. robur* (b). In comparison with control plants (b; Tukey HSD test), leaves of saplings dusted with cement PM, roadside PM, and construction PM accumulated approximately ~ 1.4-fold more coarse PM (all a). Regarding fine PM, *B. pendula* accumulated approximately 43% more (a; Tukey HSD test) than did either *T. cordata* (b) or *Q. robur* (b). In comparison with control plants (c; Tukey HSD test), saplings dusted with cement PM accumulated 1.6-fold more (a) fine PM, whereas saplings dusted with roadside PM and construction PM accumulated 1.3-fold more (b) fine PM.

A strong positive correlation was found for both *B. pendula* and *Q. robur* between wax and coarse PM. The same strong positive correlation was found in *Q. robur*, in addition to a strong positive correlation between wax and fine PM. There was no correlation between the amount of wax and _W_PM for any species. When we looked at all species together (average), we observed that the amount of epicuticular waxes was correlated with _S_PM, _W_PM, and all size fractions of PM (Table 2).

**Table 2:** Non-parametric Spearman’s correlation coefficients calculated between different categories of particulate matter (PM) and different characteristics measured in three tree species (*Betula pendula*, *Quercus robur*, and *Tilia cordata*), presented separately for individual species and for all species together. Bold values indicate *P* < 0.05.

	Total PM	Large PM	Coarse PM	Fine PM	Surface PM	In-wax PM
*Betula pendula* (*n* = 16)						
Epicuticular waxes	0.338	0.291	**0.677**	0.279	0.279	0.394
Annual increase in leader shoot length	**− 0.632**	**− 0.601**	− 0.330	− 0.465	**− 0.632**	**− 0.566**
Photosynthesis rate	**− 0.671**	**− 0.588**	**− 0.535**	**− 0.591**	**− 0.588**	**− 0.671**
Stomatal conductance	**− 0.515**	− 0.409	**− 0.650**	**− 0.594**	− 0.450	− 0.459
*Quercus robur* (*n* = 16)						
Epicuticular waxes	0.403	0.050	**0.606**	**0.585**	0.359	0.265
Annual increase in leader shoot length	− 0.192	− 0.254	− 0.328	− 0.161	− 0.267	0.025
Photosynthesis rate	− 0.497	**− 0.524**	− 0.406	**− 0.568**	**− 0.606**	− 0.432
Stomatal conductance	− 0.426	**− 0.544**	− 0.418	− 0.338	− 0.400	**− 0.618**
*Tilia cordata* (*n* = 16)						
Epicuticular waxes	0.321	0.338	0.376	0.382	0.321	0.247
Annual increase in leader shoot length	**− 0.613**	**− 0.613**	− 0.415	− 0.311	**− 0.607**	− 0.486
Photosynthesis rate	**− 0.765**	**− 0.729**	**− 0.753**	**− 0.859**	**− 0.724**	**− 0.747**
Stomatal conductance	**− 0.776**	**− 0.712**	**− 0.838**	**− 0.850**	**− 0.756**	**− 0.632**
All species (*n* = 48)						
Epicuticular waxes	**0.656**	**0.593**	**0.535**	**0.436**	**0.413**	**0.637**
Annual increase in leader shoot length	− 0.177	− 0.247	0.006	0.076	**− 0.404**	− 0.093
Photosynthesis rate	**− 0.713**	**− 0.648**	**− 0.762**	**− 0.842**	**− 0.524**	**− 0.743**
Stomatal conductance	**− 0.604**	**− 0.538**	**− 0.733**	**− 0.786**	**− 0.412**	**− 0.646**

### Efficiency of photosynthetic apparatus

**Fig. 2:**
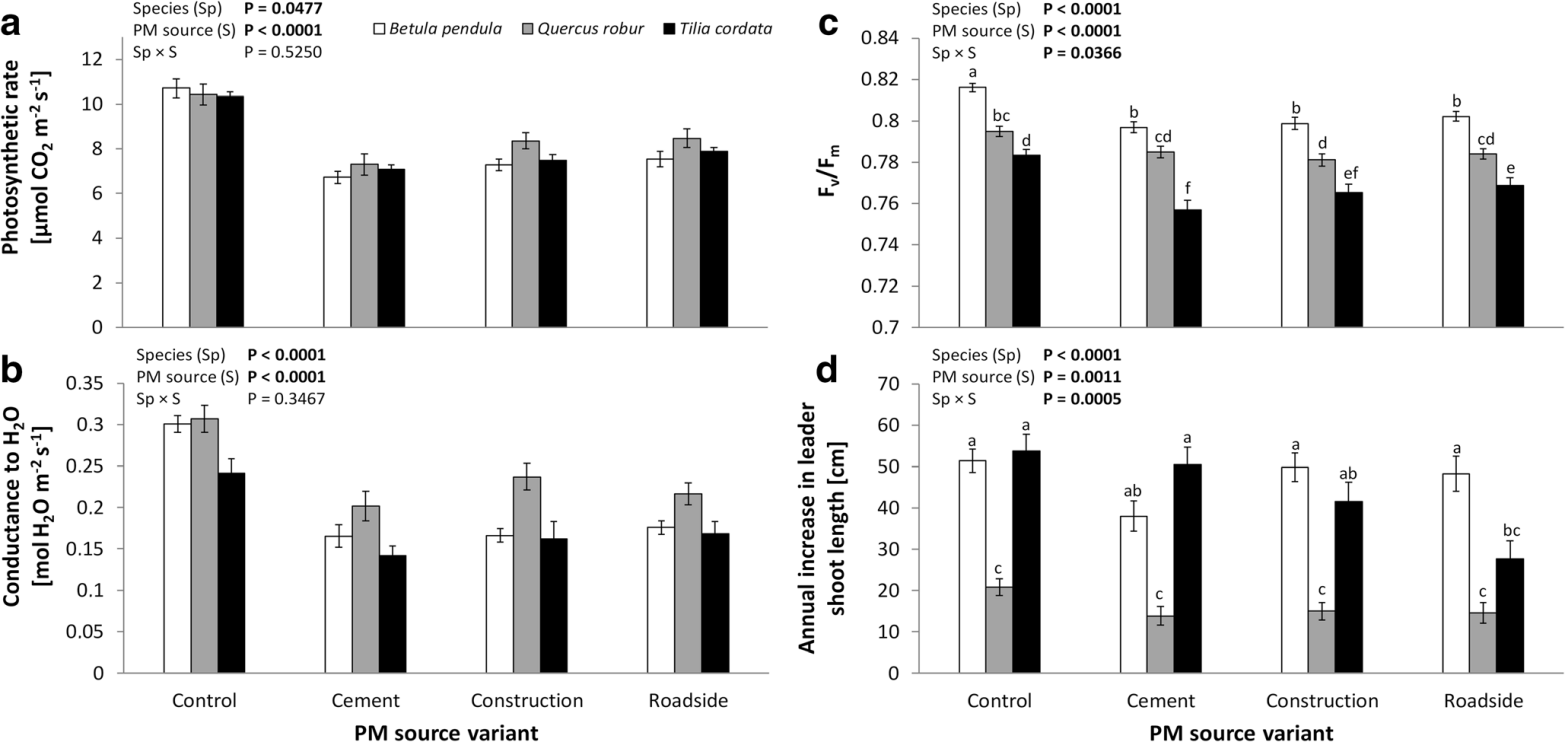
Mean rate of photosynthesis, stomatal conductance, maximum quantum efficiency of photosystem II (F_v_/F_m_), and annual increase in leader shoot length in *Betula pendula*, *Quercus robur*, and *Tilia cordata* dusted with construction particulate matter (PM), cement PM, and roadside PM. Analysis of variance (ANOVA) was used to assess the statistical significance of PM source, and Tukey’s HSD test (P = 0.05) was employed to assess the significance of differences among PM sources. Levels not connected by the same letter are significantly different. ‘Sapling’ nested in ‘plant species’ and ‘PM source’, ‘year’ (growing season), and ‘leaf’ nested within ‘sapling’ and ‘month’ were included in the model as three random effects. Bold values indicate *P* < 0.05. The data are given as means with standard errors of the mean (± SE). Sample size: (A, B) *n* = 1920 each; (C) *n* = 1152; (D) *n* = 96

For both the rate of photosynthesis (Fig. 2A) and stomatal conductance (Fig. 2B), significant effects of species and PM source were found. The rate of photosynthesis measured in *Q. robur* saplings (a; Tukey HSD test) was 7% higher than that in *B. pendula* (b), but not significantly different from that in *T. cordata* (ab), regardless of the specific PM source. The intensity of photosynthesis measured in control saplings (a; Tukey HSD test) was 49%, 36%, and 32% higher than in the cases of plants polluted by cement (c), roadside (bc), and construction PM (b), respectively. Moreover, strong negative correlations were found between photosynthesis rate and total amount of PM accumulation in *B. pendula* and *T. cordata*, but not in *Q. robur* (Table 2). Additionally, strong negative correlations between _S_PM, _W_PM, and all size fractions of PM and rate of photosynthesis were found in *B. pendula* and *T. cordata*, but only between large, fine, and _S_PM and rate of photosynthesis in *Q. robur*. When we considered all species together, we found a significant negative relationship between the photosynthesis rate and _S_PM, _W_PM, and all size fractions of PM (Table 2).

The stomatal conductance measured in *Q. robur* saplings (a; Tukey HSD test) was 27% higher than that in *B. pendula* or *T. cordata* (both b), regardless of the specific PM source. Stomatal conductance measured in control saplings (a; Tukey HSD test) was 56% higher than in the cases of PM polluted plants (all PM sources were b). Furthermore, strong negative correlations were found between stomatal conductance and total amount of PM accumulation in *B. pendula* and *T. cordata*, but not in *Q. robur* (Table 2). Moreover, strong negative correlations between _S_PM, _W_PM, and all size fractions of PM and stomatal conductance were found in *T. cordata*, but only between coarse and fine PM in *B. pendula*, and only between large PM and _W_PM in *Q. robur*. Based on data for all three species, we found a significant negative relationship between stomatal conductance and _S_PM, _W_PM, and all size fractions of PM (Table 2).

The results of chlorophyll *a* fluorescence showed significant differences among species and PM sources, and an interaction between those effects (Fig. 2C). The maximum quantum efficiency of photosystem II (F_v_/F_m_) calculated for *B. pendula* saplings (a; Tukey HSD test) was 4.5% higher and for *Q. robur* (b) was 2.3% higher than that for *T. cordata* (c). The parameter F_v_/F_m_ calculated for control saplings (a; Tukey HSD test) was 2.4%, 2.1%, and 1.7% higher than that for plants polluted by cement (c), construction (bc), and roadside PM (b), respectively. Statistical analysis showed a significant interaction between the abovementioned effects (Fig. 2C).

### Growth

Regarding annual increase in leader shoot length, we found significant effects of species and PM source, as well as interactions between the abovementioned effects (Fig. 2D). Increases in leader shoot length measured in *B. pendula* and *T. cordata* did not significantly differ in relation to the source of PM pollution (both a; Tukey HSD test), but were 181% higher than those measured in *Q. robur* saplings (b). Increases in leader shoot length measured on control saplings (a; Tukey HSD test) were 42%, 23%, and 18% higher than in the cases of plants polluted by roadside (b), cement (b), and construction PM (b), respectively. Despite a significant interaction between species and PM source, differences between the variants were not clearly marked. Moreover, significant negative correlations were found between increases in leader shoot length and total amount of PM accumulation in *B. pendula* and *T. cordata*, but not in *Q. robur* (Table 2). Additionally, in *B. pendula*, strong negative correlations were found between the increase in leader shoot length and the amount of PM in the large PM, _S_PM, and _W_PM fractions. Similar relationships also occurred for large PM, and _S_PM in *T. cordata*. Based on data for all three species, we found a significant negative correlation only between annual increase in leader shoot length and _S_PM (Table 2).

## Discussion

On the leaves of all studied species, we found two types of PM accumulation (_S_PM and _W_PM) and all three size fractions of PM. Even the control saplings, although protected from external pollution, collected a certain amount of PM, possibly originating from the exchange of air between the greenhouse and the outside environment (e.g. as a result of the exchange with the outside air during care work). Results clearly showed significant differences in PM accumulation among *B. pendula*, *Q. robur*, and *T. cordata* (Fig. 1; Table 1); however, even lower deposition on *T. cordata* leaves did not prevent the tested saplings from the adverse influence of PM on physiological processes and growth (Fig. 2). Many authors (Dzierżanowski et al. 2011; Prusty et al. 2005; Song et al. 2015) have also observed variations in dust interception ability among plant species, and they have suggested that the dust interception capacity of plants depends on their canopy shape and size, leaf phyllotaxy, and leaf surface characteristics. Many characteristics, such as surface roughness and presence of trichomes and wax layer, have been shown to increase the amount of PM on leaves (Bakker et al. 1999; Leonard et al. 2016; Popek et al. 2017b). We therefore only speculate that in *B. pendula* (which accumulated the most PM), as well as in *Q. robur* (which accumulated a medium amount of PM) with less effectiveness, PM mainly accumulated in the denser trichome layer and along the venation (Atkinson 1992; Fortini et al. 2009). The smooth leaf surface with only small tufts of hair on the abaxial side of the leaf blade of *T. cordata* is likely the main cause of its lower PM accumulation (Pigott 1991). The differences in the leaf morphological features listed in this paragraph may partly explain the differences in PM accumulation on the foliage of the study species.

In our study, carried out under controlled conditions, accumulation patterns of PM of different size fractions were consistent with the field studies’ findings of Sæbø et al. (2012). The observed pattern of accumulation of both _S_PM and _W_PM (more PM was deposited on the leaf surface than in waxes) has also been found in many other species (Dzierżanowski et al. 2011; Popek et al. 2013), important in forestry (*Acer campestre* L., *Fraxinus excelsior* L., etc.) and horticulture (*Ginkgo biloba* L., *Hedera helix* L., *Platanus* × *hispanica* Münchh., etc.). The wax layers of different plant species in most cases have different structural and chemical properties (Jeffree 2006), and in this way, the amount of accumulated PM can also differ. Generally, *B. pendula* exhibited the greatest ability to accumulate PM in our study, as well as having a greater amount of waxes on leaves than did *Q. robur* and *T. cordata*. As a consequence, a large proportion of the particulate *B. pendula* accumulated were _W_PM (its ratio of _S_PM to _W_PM was the lowest). *Betula pendula* trees studied by Sæbø et al. (2012) accumulated 83% _W_PM (total PM accumulated was used as 100%), and this was due to high levels of waxes. Based on data for all three species, we observed a significant relationship between the amount of waxes and the amount of _S_PM, _W_PM, and all size fractions of PM (Table 2). In the current investigation, all tested saplings showed a tendency for higher wax production when growing under PM pollution stress (Table 1). There is a view that the amount and structure of wax layers can be indicators of defensive response, because a greater amount of waxes deposited on leaves could be considered to be an improved barrier between plant tissue and a polluted environment (Gawrońska and Bakera 2015; Rai 2016). Our results support this view and our Hypothesis 2, because waxes appeared to play an important role in reducing PM absorption by the plant, although with varying efficiency.

Our study showed that the photosynthetic apparatus efficiency of the studied species was significantly negatively affected by the amount of PM in the case of all sapling species. Although there are rare studies of improved photosynthetic and pigment growth in some PM-polluted species (Kuki et al. 2008), the majority of reports refer to a significant reduction in these important parameters (Rai 2016). For example, iron ore dust deposition in *Eugenia uniflora* L. resulted in low values for net photosynthesis, transpiration, and chlorophyll *a* content (Neves et al. 2009). Moreover, in our previous research (Popek et al. 2017b), we have shown that *P. padus* reacts strongly to increased amounts of PM on its leaf blades by reducing its rate of photosynthesis. In our current study, photosynthesis rates measured in control saplings were higher than in the cases of polluted plants. We therefore speculate that decreases in this parameter are due to changes in the optical properties of the leaves and the decrease in radiation reaching the chlorophyll antenna, due to absorption or reflection by PM (Nanos and Ilias 2007). A similar pattern was found for the stomatal conductance of the studied species (Fig. 2B). Particles can affect the stomata, occluding the stomata and decreasing the availability of CO_2_, independent of the light phase of photosynthesis and transpiration (Naidoo and Chirkoot 2004; Nanos and Ilias 2007; Burkhardt and Grantz 2016; Siqueira-Silva et al. 2016). Our results are compatible with the data provided for *Q. coccifera* L., which indicated that the deposition of limestone dust on leaves is detrimental to photosynthesis (Vardaka et al. 1995). This detrimental effect was partially explained by the authors in terms of reduced stomatal conductance (that is, blockage of the stomata), but it may also be due to the direct toxic chemical effects or alkaline pH of the dust. Cement PM deposition could also lead to changes in density and decreases in stomata size, which is a response to pollution stress that helps to restrict the entry of dust pollutants into the plant, as well as allowing proper functioning of gas exchange (El-Khatib et al. 2012; Tiwari and Pandey 2017). Despite the shading effects of accumulated PM on the leaf blade, increased leaf temperature could also cause a decline in the efficiency of photosynthesis (Eller 1977). Temperature is one of the major environmental factors able to modulate photosynthesis and respiration processes in plants, and may vary considerably following a change of a few degrees in temperature (Robakowski et al. 2002). A strong influence of temperature on photosynthesis and productivity may therefore be expected, especially in plants with leaves dusted by roadside PM (Eller 1977).

The unfavourable effect of PM deposition on leaf blades, including stomatal closing, may be due to a decrease in the concentration of chlorophyll (Rai 2016). The photosynthetic pigments are the most likely to be damaged by air pollution (Prusty et al. 2005), and this phenomenon has been observed by many authors (Naidoo and Chirkoot 2004; Nanos and Ilias 2007; Raajasubramanian et al. 2011; Saadullah et al. 2014; Chen et al. 2015). The results of chlorophyll *a* fluorescence obtained in this work showed significant differences between dusted and control plants based on the parameter F_v_/F_m_, which described the maximum quantum efficiency of photosystem II for all tested species (Fig. 2C). A decrease in the parameter F_v_/F_m_ is a major indicator of increased deposits of PM on the leaves. Generally, photosystem II efficiency can be reduced via impaired electron transport, which follows from previously reported decreases in the rate of photosynthesis (Vardaka et al. 1995). Data obtained by Joshi and Swami (2007) indicated significant decreases in chlorophylls *a* and *b*, total chlorophyll, and carotenoids in four economically important tree species in India that were polluted by roadside PM. Additionally, decreases in chlorophyll content and increases in ascorbic acid content (an important antioxidant) were observed by Prajapati and Tripathi (2008), as a result of urban dust deposition. In general, it can be said that chlorophyll impairment can indicate a cascade of changes in a plant, especially a progressive decline in the content of important metabolites, such as proteins, amino acids, carotenoids, and total sugars (Prasad and Inamdar 1990; Rai 2016). In regard to Hypothesis 3, we fully confirmed that a large amount of PM, but set into relation of realistic PM concentrations, has a negative impact on the photosynthetic apparatus, based on the rate of photosynthesis, stomatal conductance, and F_v_/F_m_.

Of the many studies that have investigated the impact of PM on the photosynthetic apparatus, several have provided information concerning the negative effect of PM on the growth of plants (Brandt and Rhoades 1972; Gupta and Ghouse 1987; Saadullah et al. 2014). This negative impact may be due to the fact that there is likely a relationship between biochemical parameters, and plant growth and development (Saadullah et al. 2014; Rai 2016). For example, Prasad and Inamdar (1990) found that cement PM reduced plant height in dusted plants; this response to environmental pollution was likely due to decreased photosynthesis per unit leaf, and increased respiration. In the present study, despite a significant decrease in the value of physiological parameters, we did not observe a clear relationship with the growth of saplings (only a decrease in annual increase in leader shoot length in *T. cordata*, under roadside PM stress). The collected data were characterised by high variation, which did not allow for sufficiently clear statements. In addition, more data on other growth parameters would be required to exactly determine the impact of PM on tree growth. Thus, we partially confirmed Hypothesis 4, that PM has a negative impact on annual increases in leader shoot length.

In summary, our research shows that under controlled conditions research that the three studied species accumulate PM on their leaf blades, reducing the efficiency of their photosynthetic apparatus, which in a broader sense can be considered a reduction of their normal functioning and condition. None of the species studied were fully protected from excessive accumulation (e.g. due to their specific morphological and anatomical leaf traits), but variation was observed. *Quercus robur* suffered the least, with *B. pendula* (especially photosynthetic rate and stomatal conductivity) and *T. cordata* (annual increases in leader shoot length) suffering greater negative effects. The differences in PM capacity indicated that the most PM would likely accumulate on *B. pendula*, regardless of the source of the dust. We can assume that together with PM, many harmful heavy metals and polycyclic aromatic hydrocarbons were also accumulated, as observed in other studies (Popek et al. 2017a). Thus, *B. pendula* shows the best potential to enhance the quality of a PM-contaminated environment, especially due to its ability to accumulate 43% more fine PM than *Q. robur* and *T. cordata*. Like Sæbø et al. (2012) in their field study, we believe that *B. pendula* should more often be the tree species chosen for planting, because it can accumulate more pollution in urban forests and along roads than other deciduous species in Europe, such as *A. campestre*, *A. pseudoplatanus* L., *F. excelsior*, *H. helix*, and *Populus tremula* L. The low ecological requirements and widespread occurrence of *B. pendula* offer the possibility of using this species as an indicator of environmental PM contamination in many diverse habitats, and the rapid growth and large final size of this tree species suggest its use as an effective barrier to the spread of PM. As noted above, however, like the other two species studied in this work, *B. pendula* experiences significant negative effects from PM pollution. We should therefore expect reduced productivity of this species, as well as likely reduced quality of its wood as a raw material, if harvesting wood from such individuals were to be considered. Another problem that is associated with the use of *B. pendula* is the short leaf lifespan (e.g. *Q. robur* leaves live much longer), as well as the fact that PM contamination also causes faster leaf fall (Prajapati 2012). Looking at all of this more broadly, the selection of trees by architects and urban planners for planting in areas potentially affected by PM must be well-thought-out and must take into account more plant characteristics than just their capacity to accumulate dust. Among the species of trees studied in this investigation, the species most fully satisfying these conditions is *B. pendula*. It should be emphasized that the application of the dust comes from the top, i.e. deposition mostly is due to gravitational deposition, which will favour heavy particles. When transferring the results, it should be noted that aerosol deposition trees growing in field conditions will also be influenced by wind and the depositional processes of impaction and diffusion. They affect mainly the deposition of fine aerosols, and fine aerosols are most dangerous to humans. The next step in the research should be surface or biomass study of foliage. The results of these tests, e.g. in the form of allometric equitation and appropriate calculations, should allow better comparison and selection of the most useful species.
